# “Planeterranean” Diet: extending worldwide the health benefits of Mediterranean Diet based on nutritional properties of locally available foods

**DOI:** 10.1186/s12967-022-03433-4

**Published:** 2022-05-17

**Authors:** Annamaria Colao, Claudia Vetrani, Giovanna Muscogiuri, Luigi Barrea, Antonia Tricopoulou, Laura Soldati, Prisco Piscitelli, UNESCO Chair on Health Education and Sustainable Development

**Affiliations:** 1 UNESCO Chair on Health Education and Sustainable Development, Federico II University, Naples, Italy; 2 Department of Clinical Medicine and Surgery, Endocrinology Unit, Federico II University, Naples, Italy; 3 Department of Human Sciences, Pegaso Telematic University, Naples, Italy; 4 Hellenic Health Foundation, Athens, Greece; 5 University of Milan, Milan, Italy

**Keywords:** Mediterranean diet, Healthy diet, Sustainability, Local foods

## Abstract

In 2010, November 16th, the Mediterranean diet was given the recognition of UNESCO as an “Intangible Heritage of Humanity” as this dietary pattern is rooted in the preservation of tradition, land, and biodiversity. In addition, mounting evidence supported the pivotal role of the Mediterranean diet in the prevention of non-communicable diseases. Nevertheless, the application of this dietary pattern in non-Mediterranean countries is still challenging. “Planeterranean” is an attempt of the UNESCO Chair of “Health Education and Sustainable Development” to prompt each country to rediscover its own heritage and develop healthier dietary patterns based on traditional and local foods.

The topic of worldwide nutritional deficiencies in relation to locally available foods and their ability to meet nutrient requirements has been recently highlighted by scientific community [1–3]. Mediterranean Diet (MD)—acknowledged as UNESCO intangible cultural heritage of humanity since 2010—is characterized by a healthy nutritional model consisting mainly of olive oil as source of unsaturated fats, nuts, legumes, vegetables, whole grain, fresh or dried fruit, a moderate amount of fish, as well as dairy, meat, and red wine [4]. MD has shown evidence-based health benefits due to a remarkable nutritional profile resulting in a reduced prevalence of cardiovascular, metabolic or neurodegenerative diseases and cancer, representing at the same time a sustainable model of food production and consumption thanks to the use of local products (able to foster biodiversity and respect for natural resources but also cultures or traditions) [5, 6]. The challenging issue of MD transferability to non-Mediterranean countries has been already triggered [7].

Nevertheless, as UNESCO Chair on Health Education and Sustainable Development, we are aimed at assessing the possibility of promoting worldwide a healthy and sustainable dietary model based on nutritional properties of MD but implemented at local level by using the food products available in the different areas of the world.

“Planeterranean” is the name that we have conceived for this new dietary model, which would be consistent with the Sustainable Development Goals (SDGs) set by United Nations in the Agenda 2030 and with the principles of circular economy.

Actually, the vast majority of people living in urban areas present a poor diet quality and variety, with most of energy intake coming from foods with high glycaemic index (i.e., white rice and potatoes), sugar-rich and fatty ultra-processed foods (i.e., ready-to-eat foods, sugar-sweetened beverages, pastries, chips, candies, etc.). These eating habits, which are increasingly frequent also in Mediterranean countries, are known for their unfavourable effects on blood glucose homeostasis and lipid profile, becoming a major cause of the worldwide obesity epidemic (unfortunately involving also children), metabolic and cardiovascular diseases [8]. On the opposite, in every place of the world, it is possible to identify specific fruits, vegetables, legumes, wholegrain, and sources of unsaturated fats which present nutritional contents and characteristics similar to those provided by typical foods of MD, likely to have also similar health benefits for populations living far from the Mediterranean area.

In Latin America, avocado, papaya, *platanos* (green bananas), and *açaí* berries represent good sources of monounsaturated fatty acids (MUFA), micronutrients, and polyphenols [9]. Other cereals from Central Africa, i.e., tapioca/manioc and teff are thought to foster the production of short-chain fatty acids (SCFA), as it occurs for whole grain typical of MD. Moreover, Quinoa is rich in proteins and provides essential amino acids, with limited fat content (mainly consisting of oleic and linoleic acids) [10]. Canadian canola oil as well as pecans nuts contain MUFA, monounsaturated fatty acids (PUFA) and phytosterols, and have shown a great LDL-cholesterol lowering effect [11]. Such popular subtropical products as pinto beans and okra, rich in fibres and proteins, are also associated with reduced LDL-cholesterol levels and lower incidence of metabolic syndrome or cardiovascular events [12]. Sesame seeds and soy intake, traditionally used in Asia, contain bioactive compounds and antioxidant substances able to reduce hypertension, oxidative stress, insulin resistance, and inflammatory markers [13, 14].

Marine macroalgae (i.e., seaweeds and wakame) and spiruline (rich in omega-3, omega-6, PUFA and MUFA) are widely consumed in Eastern countries, representing a major source of complex polysaccharides, minerals, proteins, and vitamins, showing anticancer, antiviral, antioxidant, antidiabetic, and anti-inflammatory properties [15]. Australian Macadamia nut, Davidson’s plum, native pepper berry, finger lime, and bush tomato—rich in flavonoids, vitamins, and minerals—present antioxidant and anti-inflammatory activity and are already used as functional foods and nutraceuticals [16]. On this basis, we strongly believe that the vegetables, fruits, cereals, and unsaturated fats available in different parts of the world may be combined to design evidence-based local nutritional paradigms. The UNESCO Chair on Health Education and Sustainable Development is aimed at assuming a specific commitment in defining multiple “nutritional pyramids”—based on the foods available in different parts of the world—presenting the same nutritional properties and health benefits (as well as environmental-friendly production processes) observed for Mediterranean Diet. There is also the possibility of making directly available some vegetables typical of Mediterranean areas—taking into account the differences in terms of seasonality—or the growing of other fruit trees or plants from the other continents to the Mediterranean countries (as occurred with the kiwi). Therefore, we are searching for possible contributors to this challenging activity coming from all parts of the world, who are willing to be involved in a specific research program that will be launched trough a dedicated UNESCO Chair platform under the name of “Planeterranean”.

## Data Availability

Not applicable.

## Abbreviations

MD: Mediterranean Diet
MUFA: Monounsaturated fatty acids
PUFA: Polyunsaturated fatty acids
SCFA: Short-chain fatty acids
